# Convolutional Deep Belief Networks for Single-Cell/Object Tracking in Computational Biology and Computer Vision

**DOI:** 10.1155/2016/9406259

**Published:** 2016-10-26

**Authors:** Bineng Zhong, Shengnan Pan, Hongbo Zhang, Tian Wang, Jixiang Du, Duansheng Chen, Liujuan Cao

**Affiliations:** ^1^Department of Computer Science and Engineering, Huaqiao University, Xiamen, China; ^2^School of Information Science and Technology, Xiamen University, Xiamen, China

## Abstract

In this paper, we propose deep architecture to dynamically learn the most discriminative features from data for both single-cell and object tracking in computational biology and computer vision. Firstly, the discriminative features are automatically learned via a convolutional deep belief network (CDBN). Secondly, we design a simple yet effective method to transfer features learned from CDBNs on the source tasks for generic purpose to the object tracking tasks using only limited amount of training data. Finally, to alleviate the tracker drifting problem caused by model updating, we jointly consider three different types of positive samples. Extensive experiments validate the robustness and effectiveness of the proposed method.

## 1. Introduction

Cell and object tracking have been an active research area in computational biology [1, 2] and computer vision [3–6] with a lot of practical applications, for example, drug discovery, cell biology, intelligence video surveillance, self-driving vehicles, and robotics. Despite much progress made in recent years, designing robust cell and object tracking methods is still a challenging problem due to appearance variations caused by nonrigid deformation, illumination changes, occlusions, dense populations and cluttered scenes, and so forth. Therefore, one key component in cell and object tracking is to build a robust appearance model that can effectively handle the above-discussed challenges.

Over the years, discriminative model based appearance modeling has been popular due to its effectiveness in extrapolating from relatively small number of training samples. Most existing methods focus on two aspects to construct a robust discriminative appearance model: feature representation and classifier construction. 

*Feature Representation.* Tremendous progress has been made in feature representation for cell and object tracking. Typically, a number of cell and object tracking methods employ simple color [7] or intensity [8] histograms for feature representation. Recently, a variety of more complicated handcrafted feature representations has been applied in cell and object tracking, such as subspace-based features [9, 10], Haar features [11–13], local binary pattern (LBP) [14], histogram of gradient (HoG) [15, 16], scale invariant feature transformation (SIFT) [17], and shape features [18]. While the above handcrafted features have achieved great success for their specific tasks and data domains, they are not effective to capture the time-varying properties of cell and object appearances.

*Classifier Construction.* Designing a good classifier plays another important role in the robust appearance model. The typical classifiers include ensemble learning [19–22], structural learning [18, 23], support vector machine [24], sparse coding [25, 26], coupled minimum-cost flow [27], and semi-supervised learning [28, 29]. However, due to the fact that appearance variations are highly complex, most of these classifiers suffer from their shallow structures.

**Figure 1:**
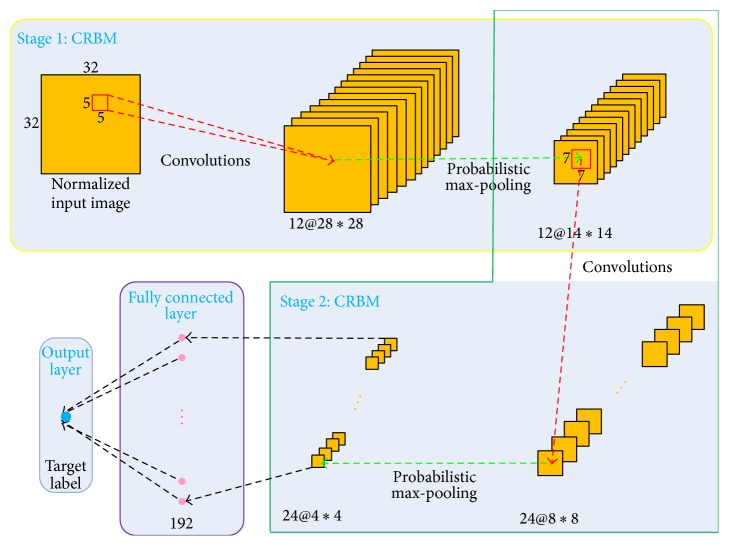
Illustration of how the proposed CDBNTracker constructs an appearance model from a convolutional deep belief network. The raw input image is fed to a 2-stage convolutional deep belief network consisting of two max-pooling CRBMs and one fully connected layer. Each CRBM contains a filter bank layer and a probabilistic max-pooling layer, respectively. The outputs of the second stage are followed by one fully connected layer with 192 units.

In this paper, inspired by the remarkable progress in deep learning [30–34] for big data analysis [35], we propose a robust cell and object tracking method (termed CDBNTracker) that relies on convolutional deep belief networks (CDBNs) to address both limitations raised from handcrafted feature and shallow classifier designs. As shown in Figure 1, our CDBNTracker is built upon the CDBNs trained from raw pixels, which is composed of two convolutional restricted Boltzmann machines (CRBMs) and one fully connected layer. To the best of our knowledge, it is the first time to apply DBN-like network architectures into cell and object tracking.

The CRBMs are stacked on top of one another, each of which contains a filter bank layer and a probabilistic max-pooling layer, respectively. With end-to-end training, CDBNTracker automatically learns hierarchical features in a supervised manner, making it extremely discriminative in appearance modeling. We further propose a transferring strategy to better reuse the pretrained CDBN features on the cell and object tracking tasks. This allows the CDBNTracker to learn cell or object-specific feature representations.

Last but not least, we propose a systematic and heuristic solution to alleviate the tracker drifting problem for the CDBNTracker. In particular, we classify the positive samples into three categories to update the CDBN-based appearance models, that is, ground-truth samples (nonadaptive samples obtained in the first frame), long-term samples (moderately adaptive samples obtained in the most recent frames), and short-term samples (highly adaptive samples collected in the current frame). The advantages of our CDBNTracker are threefold.

(1) Our CDBNTracker follows the cutting-edge deep learning framework. And the proposed CDBNTracker differs from the recent deep learning-based trackers by using multilayer CDBNs with local tied weights to reduce the model complexity under the scarcity of training samples. Furthermore, we transfer generic visual patterns as good initialization in our tracker to alleviate the “the first frame labeled” problem.

(2) We develop a new model update strategy to effectively alleviate the tracker drift. In addition to short-term and first frame information, long-term information is selectively memorized for updating the current model state to alleviate the abrupt appearance changes.

(3) Different from most previous trackers which use handcrafted features and shallow models, our CDBNTracker is online trained with a multilayer CDBN in a supervised manner which is more discriminative and descriptive.

The rest of the paper is organized as follows. An overview of the related work is given in Section 2.  Section 3 introduces how to learn a data-driven cell or object appearance model from a CDBN. The detailed tracking method is then described in Section 4. Experimental results are given in Section 5. Finally, we conclude this work in Section 6.

## 2. Related Work

Over the past decades, a huge amount of cell and object tracking methods have been proposed [1–6]. Since the proposed tracking method focuses on utilizing deep learning to construct robust appearance models for cell and object tracking, in this section, we firstly review online generative and discriminative tracking methods. Then, cell tracking methods are also briefly introduced. Finally, we discuss the current progress using deep learning for the cell and object tracking research.

### 2.1. Online Cell and Object Tracking

#### 2.1.1. Generative Models

Generative tracking models describe the cell and object appearances via a statistical model using the reconstruction errors. Some representative methods include mean shift-based tracker [7], integer programming-based tracker [8], PCA-based tracker [9], sparse coding-based trackers [25, 26], GMM-based tracker [36], multitracker integration [37], and structured learning-based tracker [18]. While generative tracking methods usually succeed in less complex scenes due to the richer appearance models used, they are prone to fail in complex scenes without considering the discriminative information between the foregrounds and backgrounds.

#### 2.1.2. Discriminative Models

On the other hand, discriminative tracking models typically view cell and object tracking as a binary classification task. Thus, they aim to explicitly learn a classifier which can discriminate the cell or object from the surrounding backgrounds. In [38], an ensemble learning-based tracker is proposed, in which a group of weak classifiers is adaptively constructed for object tracking. In [11], an online boosting-based tracker is proposed for object tracking. Grabner and Bischof [11] extend a boosting algorithm for online discriminative tracking. However, online learning-based trackers is prone to the tracker drifting problem. Recently, various discriminative tracking methods have been proposed to alleviate the drifting problem. Using an anchor assumption (i.e., the current tracker does not stray too far from the initial appearance model), Matthews et al. [39] develop a partial solution for the template-based trackers. In [20], a semi-supervised boosting algorithm is applied to online object tracking by using a prior classifier. It is obvious that the semi-supervised boosting-based tracker is not robust to very large changes in appearance. In [28], Babenko et al. present a multiple instance boosting-based tracking method. Hare et al. [12] employ an online kernelized structured output support vector machine for object tracking. In [23], an online structured support vector machine-based tracker is proposed. Duffner and Garcia [29] use a fast adaptive tracking method to track nonrigid objects via cotraining. A number of attempts have been made to apply transfer learning to object tracking [40, 41]. However, they may be limited by using handcrafted features which cannot be simply adapted according to the new observed data obtained during the tracking process.

#### 2.1.3. Cell Tracking Methods

Recently, with the rapid development of cell and computational biology, several cell tracking methods have been proposed. In [8], Li et al. employ integer programming for multiple nuclei tracking in quantitative cancer cell cycle analysis. In [18], Lou et al. propose an active structured learning method for multicell tracking, in which a compatibility function (i.e., global affinity measure) is designed to associate hypotheses and score. In [27], Padfield et al. present a cell tracking method via coupling minimum-cost flow for high-throughput quantitative analysis.

### 2.2. Deep Learning for Cell and Object Tracking

Due to the powerful representation abilities, deep learning [33] has recently drawn more and more attention in computational biology, medical imaging analysis [42], computer vision [32, 43], speech recognition [31], natural language processing, and so forth. Deep belief networks [44], autoencoders, and convolutional neural networks [32] are the three representative deep learning methods for computational biology and computer vision.

Despite the fact that tremendous progress has been made in deep learning, only a limited number of tracking methods using the feature representations from deep learning have been proposed so far [42, 45–50]. In [46], a convolutional neural network-based tracking method is proposed for tracking humans. However, once the model is trained, it is fixed during tracking due to the features being learned during offline training. In order to handle the left ventricle endocardium in ultrasound data, Carneiro and Nascimento [42] fuse multiple dynamic models and deep learning architecture in a particle filtering framework. In [51], without using the fully connect layers in convolutional neural networks, a fully convolutional neural network is proposed for object tracking. In [47], a convolutional neural network-based tracking method is presented, in which a pretrained network is transferred to an interested object. Ma et al. [48] combine the pretrained VGG features [52] and correlation filters to improve location accuracy and robustness in object tracking. In [49], a multidomain convolutional neural network-based tracking method is proposed. In [50], Chen et al. propose a convolutional neural network-based tracking method, which transfers the pretrained features from a convolutional neural network to the tracking tasks. Compared to Chen's method using a convolutional neural network, our CDBNTracker explores a different deep learning algorithm (i.e., a convolutional deep belief network, CDBN) for single-cell/object tracking. Instead of using convolutional neural networks, an autoencoder-based tracking method [45] is proposed, in which the generic image features are firstly learned from an offline dataset and then transferred to a specific tracking task.

In this paper, we focus on how to construct an effective CDBN-based appearance model for discriminative single-cell and object tracking in cell biology and computer vision, respectively. To the best of our knowledge, it is the first time to apply DBN-like network architectures to single-cell and object tracking.

## 3. Object Appearance Model

In this section, we address the problem of how to learn a data-driven appearance model from a CDBN.

### 3.1. CRBM and CDBN

The CDBN [43] is a hierarchical generative model composed of one visible (observed) layer and many hidden layers, that is, several CRBMs stacked on top of one another. A statistical relationship between the units in the lower layer is learned by each hidden layer unit; the higher layer representations tend to become more complex and abstract. Following the notations of Lee et al. [43], we briefly review the CRBM and CDBN.

The CRBM is an extension of the RBM which fully connects the hidden layer and visible layer. To capture the 2D structural of image and incorporate translation invariance, the CRBM shares the weights between the hidden units and the visible units among all locations in the hidden units. The CRBM consists of a visible (input) layer and a hidden layer. In this paper, we use real-valued visible units *v* ∈ *R* ^*n*_*V*_×*n*_*V*_^ and binary-valued hidden units *h* ∈ {0,1}^*n*_*H*_×*n*_*H*_^. Denote *W* ^*k*^ ∈ *R* ^*n*_*W*_×*n*_*W*_^ as the *k*th convolution filter weight between a hidden unit and the visible unit; *b*_*k*_ ∈ *R* as a bias variable shared among hidden units and *c* ∈ *R* as a visible bias shared among visible units. The energy function of the probabilistic max-pooling CRBM with real-valued visible units can then be defined as

(1) Ev,h=12∑i,j=1nVvij2−∑k=1 K∑i,j=1 nH∑r,s=1nWhijkWrskvi+r−1,j+s−1−∑k=1Kbk∑i,j=1nHhijk−c∑i,j=1nVvij,s.t.  ∑i,j∈Bahijk≤1,  ∀k,a,

where *K* is the number of convolution filters and *B*_*a*_ = {(*i*, *j*)∣*h*_*ij*_^*k*^  belonging  to  the  block  *a*} is a *C* × *C* block of locally neighboring hidden units *h*_*ij*_^*k*^ that are pooled to a pooling unit *p*_*a*_^*k*^. It should be noted that probabilistic max-pooling enables the CRBM to incorporate max-pooling-like behavior, while allowing probabilistic bottom-up and top-down inference [43]. The conditional probability distributions can be calculated as follows:

(2) Phijk=1 ∣ v=exp⁡Ihijk1+∑i′,j′∈Baexp⁡Ihi′j′k,Pvij ∣ h=N∑kWk∗fhkij+c,1,Ppak=0 ∣ v=11+∑i′,j′∈Baexp⁡Ihi′j′k,

where $I(h_{ij}^{k})=({\tilde{W}}^{k}{*}_{v}v{)}_{ij}+b_{k}$, *∗*_*f*_ is a full convolution, *∗*_*v*_ is a valid convolution, and ${\tilde{W}}_{ij}^{k}=W_{n_{W}-i+1,n_{W}-j+1}^{k}$.

Typically, the CRBM is highly overcomplete due to the fact that the hidden layer of the CRBM contains *K* groups of units, each roughly with size of the visible layer (input image). To avoid the risk of learning trivial solutions by the CRBM, a sparsity penalty term is added to the log-likelihood objective function of the training data. Consequently, each hidden unit group has a mean activation close to a small constant. Finally, after the greedy and layer-wise training, we stack the CRBMs to form a CDBN.

### 3.2. Learning Cell and Object Appearance Models from CDBNs

**Figure 2:**
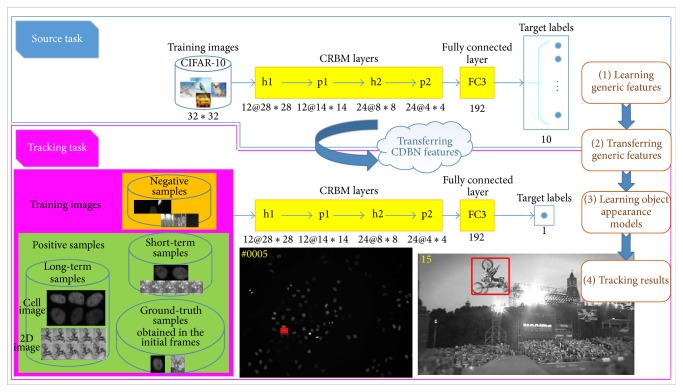
Learning object appearance models by transferring the CDBN features. First, the CDBN is pretrained on the source task (CIFAR-10 classification, top row). Then, the pretrained parameters of the internal layers of the CDBN (h1–FC3) are then transferred to the tracking task (bottom row). To achieve the transfer and construct the cell and object appearance models, we remove the output layer with 10 units and add an output layer with one unit. Furthermore, to alleviate the drifting problem, we treat training samples differently to update the cell and object appearance models.

In this paper, we view object tracking as an online transfer learning problem and use the CDBN to construct the cell and object appearance model due to its capacity for automatically learning a hierarchical feature representation. As shown in Figure 2, the key idea is to use the internal CDBN features as a generic and middle-level image representation, which can be pretrained on one dataset (the source task here CIFAR-10 [53]) and then reused on the tracking tasks.

**Figure 3:**
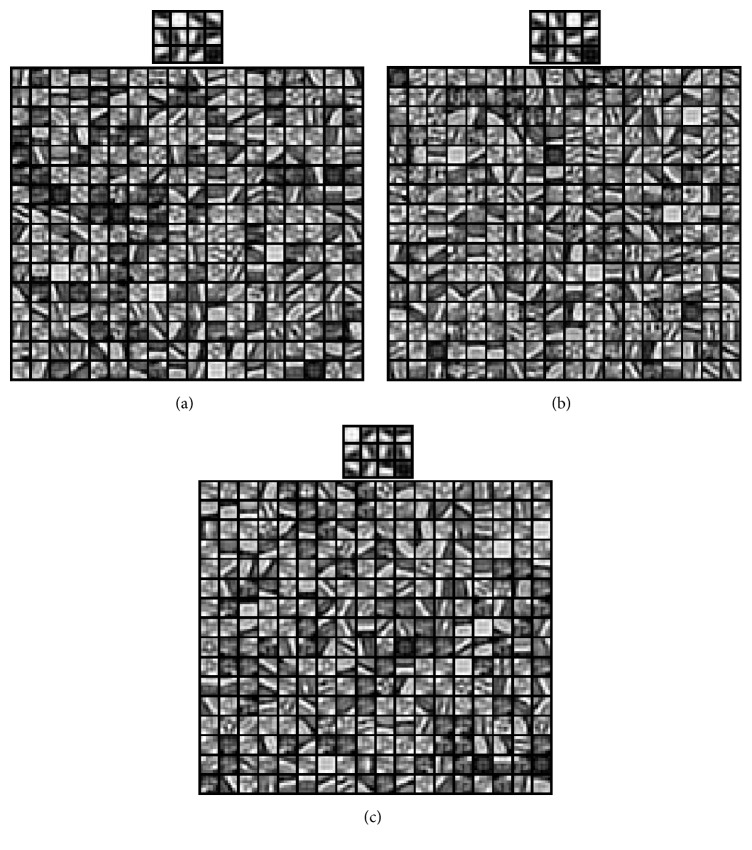
(a) The pretrained filters in first (top) and second (bottom) CRBM layer learned from CIFAR-10 natural images. (b) The fine-tuned filters in first (top) and second (bottom) CRBM layer learned from the training data of motorRolling sequence [6]. (c) The fine-tuned filters in first (top) and second (bottom) CRBM layer learned from the training data of Mitocheck sequence [54].

More specifically, for the source task, we pretrain a CDBN with two CRBM layers followed by one fully connected layer from the CIFAR-10 natural image dataset [53]. The CIFAR-10 dataset is a labeled subset of the 80 million tiny images, containing 60,000 images and ten classes. Each CRBM layer is composed of a hidden and pooling layer. The first CRBM layer consists of 12 groups of 5*∗*5 convolution filters, while the second CRBM layer consists of 288 groups of 7*∗*7 convolution filters. The pooling ratio is set to 2 for each pooling layer. The target sparsity for the first and second CRBM layer is set as 0.003 and 0.005, respectively. The fully connected layer FC3 has 192 units. The output layer has size 10 equal to the number of target categories. It can be seen from Figure 3(a) that the learned filters in first CRBM layer (top) are oriented and localized edge filters, while the learned filters in second CRBM layer (bottom) selectively respond to contours, corners, angles, and surface boundaries in the images.

After pretraining on the source task, the parameters of layers h1, p1, h2, p2, and FC3 are first transferred to the tracking task. Then, we remove the output layer with 10 units and add an output layer with one unit. Finally, the newly designed CDBN is retrained (fine-tuned) on the training data from a specific tracking task to learn a cell or object appearance model. This simple yet effective transferring schema enables the proposed CDBNTracker to tackle the domain changes in training tasks. To empirically illustrate the efficacy of the transfer, we check the fine-tuned filters trained on the training data from a specific tracking task.  Figure 3(b) shows the fine-tuned filters trained on the training data from the first frame of the motorRolling sequence [6].  Figure 3(c) shows the fine-tuned filters trained on the training data from the first frame of the Mitocheck sequence [54]. It can be seen from both Figures 3(b) and 3(c) that, in addition to edge, corner, and junction detectors, the transferred CDBN also adaptively learns different and complicated features according to the newly observed data.

## 4. Single-Cell and Object Tracking via CDBNs (CDBNTracker)

In this section, we present a single-cell and object tracking method, in which the CDBN-based appearance model is effectively incorporated into a particle filtering framework. The particle filtering framework consists of two key components.

(1) A dynamic model *p*(*x*_*t*_∣*x*_*t*−1_) generates candidate samples based on previous particles. In this paper, the dynamic model between two consecutive frames is assumed to be a Gaussian distribution: *p*(*x*_*t*_∣*x*_*t*−1_) = *N*(*x*_*t*_; *x*_*t*−1_, ∑), where ∑ denotes a covariance matrix and *x*_*t*_ = (*p*_*t*_^*x*^, *p*_*t*_^*y*^, *w*_*t*_, *h*_*t*_) denotes the cell or object state parameters composed of the horizontal coordinate, vertical coordinate, width, and height, respectively.

(2) An observation model *p*(*y*_*t*_∣*x*_*t*_) calculates the similarity between candidate samples and the cell or object appearance model. In this paper, the proposed CDBN-based appearance model is used to estimate the score of the likelihood function *p*(*y*_*t*_∣*x*_*t*_).

To capture the appearance variations, the observation model (i.e., the CDBN-based appearance model) needs to be updated over time. Therefore, to alleviate the tracker drifting problem, we classify the positive samples into three categories: ground-truth samples (nonadaptive samples obtained in the first frame), long-term samples (moderately adaptive samples obtained in the most recent frames via FIFO schema), and short-term samples (highly adaptive samples collected in the current frame). We assume the ground-truth set of positive samples obtained in the first frame to be *s*_*g*_^+^ = {*x*_1,*i*_^+^}_*i*=1_^*N*_1_^+^^. The long-term set of positive samples obtained in the most recent frames is denoted as *s*_*lt*_^+^ = {*x*_*t*−*i*_^+^}_*i*=1_^*T*^, where *T* is the buffer size of temporal sliding window. The sets of negative samples and short-term positive samples collected in the current frame are denoted as *s*_*t*_^−^ = {*x*_*t*,*i*_^−^}_*i*=1_^*N*_*t*_^−^^ and *s*_*t*_^+^ = {*x*_*t*,*i*_^+^}_*i*=1_^*N*_*t*_^+^^, respectively. At each frame *t*, we update the CDBN-based appearance model using *s*_*g*_^+^, *s*_*lt*_^+^, *s*_*t*_^+^, and *s*_*t*_^−^.

Finally, a summary of our CDBN-based tracking method for single-cell and object tracking is described in Algorithm 1.

**Algorithm 1 (single-cell and object tracking via learning and transferring CDBN).**

 

*Initialization*

(1) Pretrain a CDBN on the CIFAR-10 dataset.
(2) Acquire manual labels in the first frame. Collect the ground-truth set of positive samples *s*_*g*_^+^ and negative samples *s*_1_^−^.
(3) Resize each positive/negative image patch to 32*∗*32 pixels.
(4) Construct the CDBN-based appearance model via fine-tuning and transferring the pre-trained CDBN using *s*_*g*_^+^ and *s*_1_^−^.
(5) Initialize the particle set {*x*_1_^*i*^, *w*_1_^*i*^}_*i*=1_^*N*_1_^ at time *t* = 1, where *w*_1_^*i*^ = 1/*N*_1_, *i* = 1,…, *N*_1_
(6) Set the maximum buffer size *T* for long-term positive samples *s*_*lt*_^+^.

*For t* = 2* to the End of the Video*

(1) *Prediction*: for *i* = 1,…, *N*_1_, generate *x*_*t*_^*i*^ ~ *p*(*x*_*t*_∣*x*_*t*−1_^*i*^)
(2) *Likelihood evaluation*: for *i* = 1,…, *N*_1_, let *w*_*t*_^*i*^ = *w*_*t*−1_^*i*^ *p*(*y*_*t*_∣*x*_*t*_^*i*^).
(3) Determine the optimal object state *x*_*t*_^*∗*^ as the particle with the maximum weight.
(4) *Resample*: Normalize the weights and compute the covariance of the normalized weights. If this variance exceeds one threshold, then *β*_*j*_ ~ {*w*_*t*_^*i*^}_*i*=1_^*N*_1_^ and replace {*x*_*t*_^*i*^, *w*_*t*_^*i*^}_*i*=1_^*N*_1_^ with {*x*_*t*_^*β*_*j*_^, 1/*N*_1_}_*j*=1_^*N*_1_^.
(5) *Update*:
  (5.1) Set short-term positive samples *s*_*t*_^+^ at time *t* as the image patches having the 10 highest confidences (estimated by the likelihood evaluation).
  (5.2) Select negative samples *s*_*t*_^−^ at time *t*.
  (5.3) Update the long-term set of positive samples *s*_*lt*_^+^ = *s*_*lt*_^+^ ∪ {*x*_*t*_^*∗*^}.
  (5.4) If the size of *s*_*lt*_^+^ is larger than *T*, then *s*_*lt*_^+^ is truncated to keep the last *T* elements.
  (5.5) Update the CDBN-based appearance model based on *s*_*g*_^+^, *s*_*lt*_^+^, *s*_*t*_^+^ and *s*_*t*_^−^.

*End For*

## 5. Experiments

In this section, we first introduce the setting of our experiments. Then, we test the proposed CDBNTracker (CDBN-10-2), which has two CRBM layers followed by one fully connected layer and is pretrained on the CIFAR-10 dataset, the Mitocheck dataset [54], and CVPR2013 tracking benchmark [6], respectively. The Mitocheck dataset from the Mitocheck project [54] is a time-lapse microscopic image sequence. The Mitocheck sequence contains higher cell density, larger intensity variability, and illumination variations. The CVPR2013 tracking benchmark contains 50 fully annotated image sequences. Each image sequence is tagged by a number of attributes indicating the presence of different challenging aspects, such as illumination variation, scale variation, occlusion, deformation, and background clutters. To show the advantage of the CDBN-10-2 over the other competing trackers, we compare it with some state-of-the-art tracking methods including a related deep learning tracker (DLT) [45]. Moreover, the efficacy of different positive samples is empirically evaluated by a carefully designed experiment. Finally, to examine the impact of the different training data and CDBN architecture, we evaluate the performance of the proposed CDBNTracker as the amount of training data and the number of CRBM layers in CDBN grow.

### 5.1. Experiment Setting

The proposed CDBN-10-2 is implemented in Matlab on a HP Z800 workstation with an Intel® Xeon® E5620 2.40 GHz processor and 12 G RAM. The number of particles in particle filtering is set to 1,000. Each image observation of the target object is normalized to a 32*∗*32 patch. The buffer size of temporal sliding window is set as 25. To train the CDBN, we adopt stochastic gradient descent with momentum. In each frame, the number of epochs needed to train the CDBN is 500. The learning rate and momentum are set as 1*e* − 1 and 0.5, respectively. The average processing speed is about 5 fps at the resolution of 320*∗*240 pixels without using GPUs. Consequently, the proposed CDBN-10-2 can achieve real-time processing speed if the GPUs (e.g., tesla k40) are used. The main memory cost is from the number of parameters in the proposed CDBN model. However, the CDBN shares weights among all locations in an image. Thus, the number of parameters in our CDBN model is significantly reduced (to only 6.9*∗*10^4^). We only need a small-scale dataset (e.g., CIFAR-10 with 60,000 images) to pretrain our CDBN model, which can then be effectively transferred to the tracking tasks. The proposed CDBN model can obtain a better performance if we use other large-scale datasets for initialization (e.g., Caltech-256 or ImageNet). In our experiments, if the memory space of one parameter is one byte in Matlab, we find the memory cost is about 6.9*∗*10^4^/1024 = 70 KB. We use the same parameters for all of the experiments.

For performance evaluation, we test the proposed CDBN-10-2 on the Mitocheck dataset [54] and CVPR2013 tracking benchmark, respectively. In the CVPR 2013 tracking benchmark, 30 publicly available trackers are evaluated. We follow the protocol used in the benchmark, in which the evaluation is based on two different metrics: the precision plot and success plot. The precision plot shows the percentage of frames whose estimated location is within the given threshold distance of the ground truth, and a representative precision score (threshold = 20 pixels) is used for ranking. Another metric contains the overlap precision over a range of thresholds. The overlap precision is defined as the percentage of frames where the bounding box overlap exceeds a given threshold varied from 0 to 1. In contrast to the precision plot, the trackers are ranked using the area under curve (AUC) in the success plot. In addition, we compare the CDBN-10-2 against the deep learning-based tracker (DLT) of Wang and Yeung [45].

### 5.2. Comparison with Other Trackers on the CVPR2013 Tracking Benchmark

#### 5.2.1. Quantitative Evaluation

**Figure 4:**
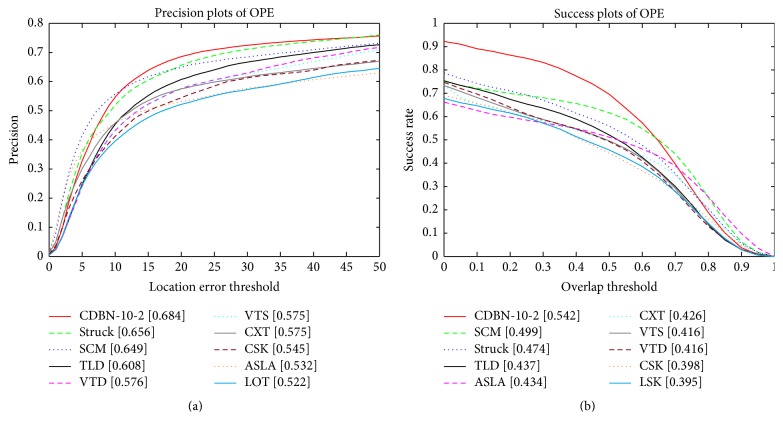
The precision and success plots of quantitative comparison for the 50 sequences in the CVPR2013 tracking benchmark [6]. The performance score of each tracker is shown in the legend. The proposed CDBN-10-2 (in red) obtains better or comparable performance against state-of-the-art tracking methods.

The quantitative comparison results of all the trackers are listed in Figure 4 where only the top 10 trackers are shown for clarity. The values in the legend of the precision plot are the relative number of frames in the 50 sequences where the center location error is smaller than a threshold of 20 pixels. The values in the legend of the success plot are the AUC. In both the precision and success plots, the proposed CDBN-10-2 is the state-of-the-art compared to all alternative methods. Our CDBN-10-2 outperforms Struck by 2.8% in mean distance precision at the threshold of 20 pixels, while it outperforms SCM by 4.3% with the AUC. The robustness of our CDBN-10-2 lies in the hierarchical and deep structure-based appearance model which is discriminatively trained online to account for each variation.

#### 5.2.2. Temporal and Spatial Robustness Evaluation

**Figure 5:**
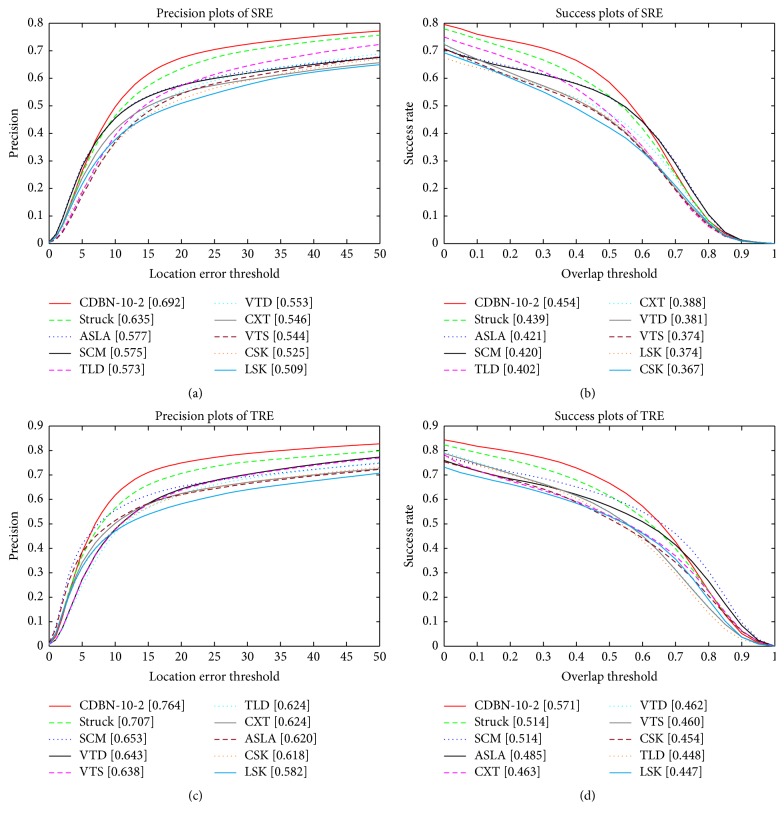
The precision and success plots for TRE and SRE. The proposed CDBN-10-2 (in red) achieves comparable performance in all the evaluations.

It is known that a tracker may be sensitive to initialization. To analyse a tracker's robustness to initialization, we follow the evaluation protocol proposed in [6] by perturbing the initialization temporally (referred to as temporal robustness, TRE) and spatially (referred to as spatial robustness, SRE). For TRE, each sequence is partitioned into 20 segments, whereas, for SRE, 12 different bounding boxes are evaluated for each sequence. The precision and success plots for TRE and SRE are shown in Figure 5. The proposed CDBN-10-2 performs favorably compared to other trackers on the temporal and spatial robustness evaluation.

#### 5.2.3. Attribute-Based Evaluation

The object appearance variations may be caused by illumination changes, occlusions, pose changes, cluttered scenes, moving backgrounds, and so forth. To analyse the performance of trackers for each challenging factor, the benchmark annotates the attributes of each sequence and constructs subsets with 11 different dominant attributes, namely,* illumination variation, scale variation, occlusion, deformation, motion blur, fast motion, in-plane rotation, out-of-plane rotation, out-of-view, background clutter*,* and low resolution*. We perform a quantitative comparison with the 30 state-of-art tracking methods on the 50 sequences annotated with respect to the aforementioned attributes. Due to space limitation, we show the representative success scores of SRE for different subsets divided based on main variation of the target object in Table 1. As we can see, the proposed CDBN-10-2 performs favorably on the 11 attributes.

#### 5.2.4. Qualitative Evaluation

**Figure 6:**
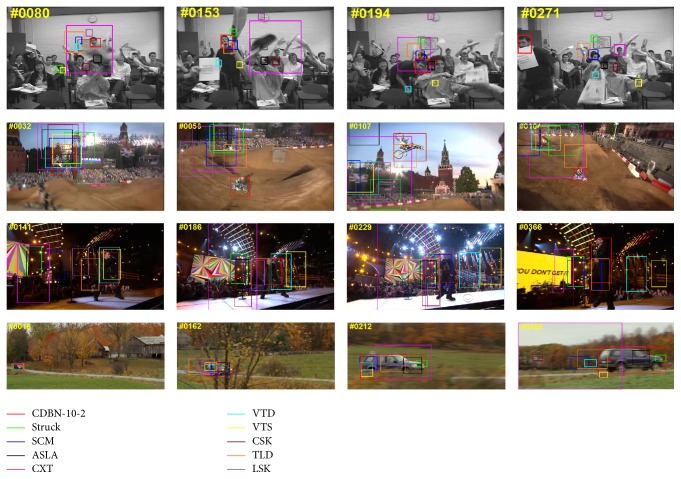
Qualitative comparison on several sequences from [6], that is, the freeman4, motorRolling, singer2, and carScale sequence, respectively.

**Figure 7:**
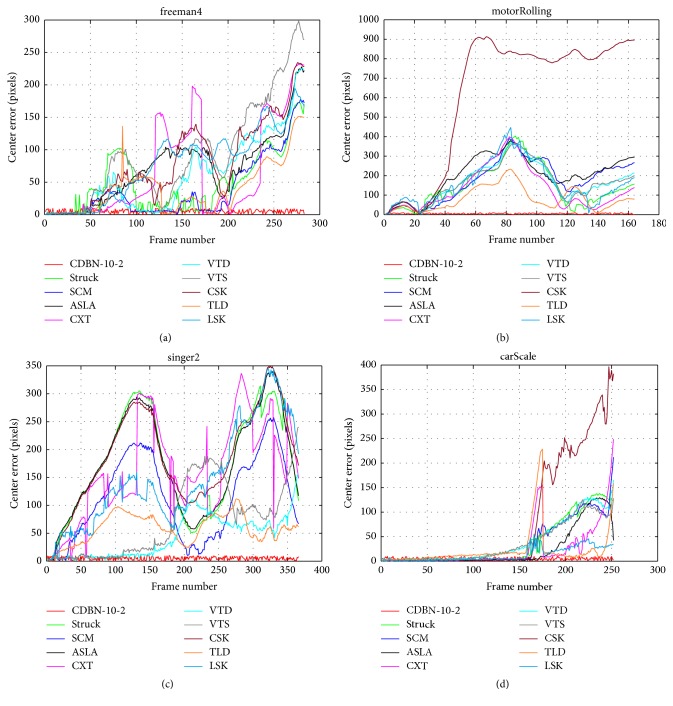
Quantitative comparison on the center distance error per frame for several sequences from [6].

Qualitative comparison with the top 10 trackers (on four typical sequences) is shown in Figure 6. Meanwhile, for more close-view evaluation, we show the corresponding examples of the center distance error per frame in Figure 7 with the top 10 trackers compared, which show that our method can transfer the pretrained CDBN features to the specific target object well.

Recall that the pretrained CDBN is learned entirely from natural scenes, which are completely unrelated to the tracking task. However, according to the overall tracking results, the proposed CDBN-10-2 outperforms the competing methods. It implies that our method can construct robust object appearance models by effectively learning and transferring the highly general CDBN features.

#### 5.2.5. Comparison with DLT [45]

To show the advantage of the CDBN-10-2 over other competing trackers based on deep learning, we compare it with the DLT [45]. According the experimental results given in [55], DLT achieves a precision of 0.452 at the threshold of 20 pixels and an AUC of 0.443 on the CVPR 2013 tracking benchmark. Although the DLT has shown good performance in several scenarios, it does not exploit the label information to learn features from the denoising autoencoder and can hardly work well in cluttered background. The proposed CDBN-10-2 outperforms DLT by 23.2% in mean distance precision at the threshold of 20 pixels, while it outperforms it by 9.9% in AUC. This is because the proposed CDBN-10-2 can effectively learn the appearance changes of the target while preserving the ability to discriminate the target from the background via combining the offline and online discriminative learning.

### 5.3. Efficacy of Different Positive Samples

**Figure 8:**
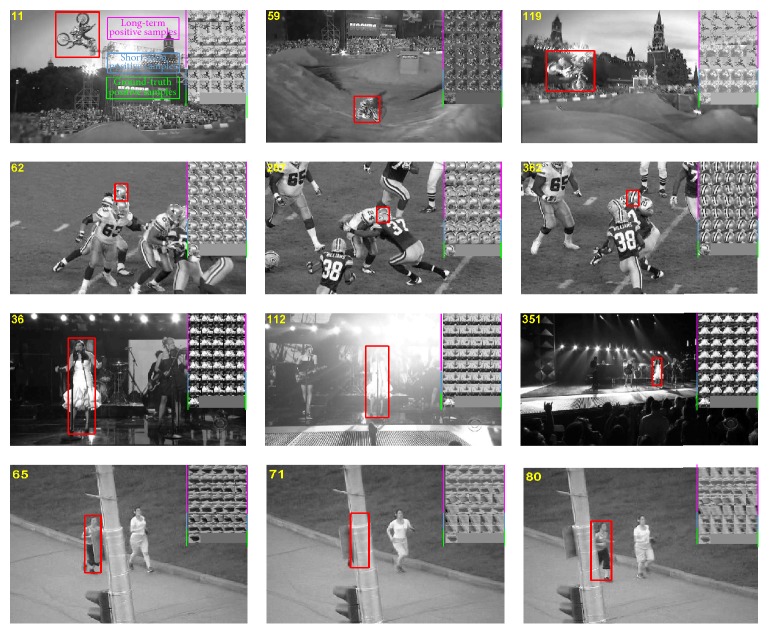
Illustration of updating process for the positive samples on several sequences from [6]. Red rectangles represent the bounding boxes of the target objects. The different positive samples are shown in the upper right corner of each image. The first row to the fifth row contain the long-term positive samples which are moderately adaptive. The sixth and seventh row contain the short-term positive samples which are highly adaptive. The last row contains the ground-truth positive samples obtained in the first frame.

One big advantage of the proposed CDBN-10-2 lies in that the positive samples are classified into three categories to capture the appearance variations while alleviating the drifting problem. To verify this advantage, we check the updating process for the positive samples and give several examples in Figure 8. The motorRolling sequence on the first row suffers from large pose and lighting variations. The football sequence on the second row contains a player moving in front of a clutter background. The singer1 sequence on the third row is captured by a PTZ camera and has large illumination changes. The jogging sequence on the fourth row suffers from short-term occlusions, pose, and appearance changes. As shown in Figure 8, it is obvious that the proposed CDBN-10-2 can effectively exploit ground-truth, long-term, and short-term positive samples to incrementally update the CDBN-10-2 to capture object appearance changes while alleviating the drifting problem.

### 5.4. The Impact of Different Training Data and CDBN Architecture

Since the proposed CDBN-10-2 consists of two CRBM layers followed by one fully connected layer and is pretrained on the CIFAR-10 dataset [53], the following questions arise: (1) why the common object recognition dataset is effective for object tracking, even though the dataset does not contain the target objects? (2) Whether the proposed CDBNTracker will continue to improve as data or the number of CRBM layers in CDBN grows? To answer these two questions, we investigate the performance of the proposed CDBNTracker as the amount of training data and the number of CRBM layers in CDBN grow.

Specifically, we first study two simple variations to the CDBN-10-2, namely, CDBN-100-2 and CDBN-tiny-2. They share the same topology of CDBN-10-2 but they are pretrained on either CIFAR-100 or tiny datatset [53]. CIFAR-100 is just like the CIFAR-10, except it has 100 classes containing 600 images each. From the 79 million tiny images, we randomly sample 202,932 images to pretrain the CDBN-tiny-2. Then, we pretrain a CDBNTracker with three CRBM layers followed by one fully connected layer from the CIFAR-10. This version of the CDBNTracker is denoted by CDBN-10-3.

**Figure 9:**
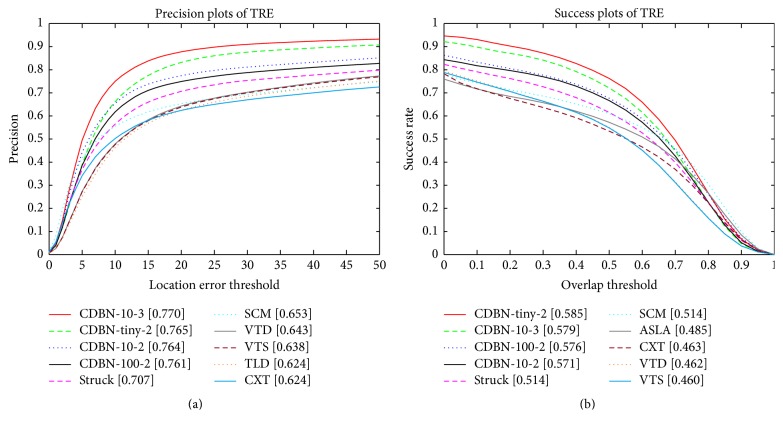
We compare the performance of the proposed CDBNTrackers (e.g., CDBN-10-2, CDBN-100-2, CDBN-tiny-2, and CDBN-10-3) as the amount of training data and the number of CRBM layers in CDBN grow.

Due to space limitation, we only show the precision and success plots for TRE on the CVPR2013 tracking benchmark in Figure 9. Obviously, the performance of the proposed CDBNTracker continues to improve as data or the number of CRBM layers in CDBN grows. Moreover, although the CDBN is trained offline for other purpose (e.g., object recognition), the proposed CDBNTracker can perform well for the tracking task by using the internal CDBN features as a generic and middle-level image representation. We conjecture that it is because the CDBN features are more effective to represent middle-level concept of target than hand-crafted ones.

### 5.5. Experimental Results on the Mitocheck Cell Dataset

**Figure 10:**
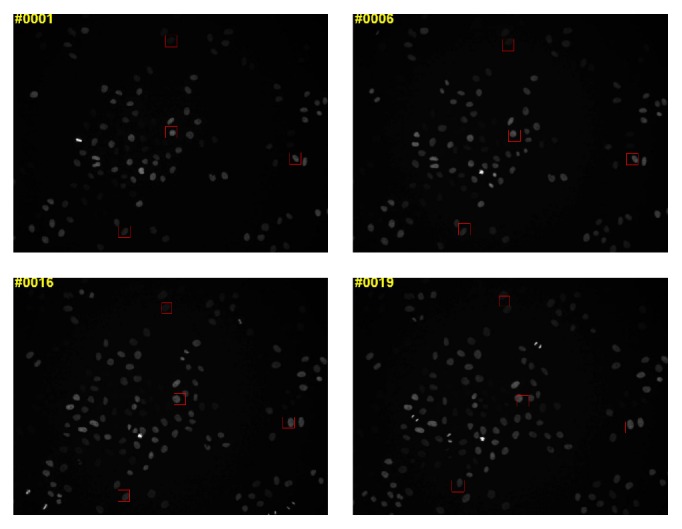
Qualitative comparison on a single-cell from the Mitocheck dataset [54].

The qualitative single-cell tracking results of our method on a single-cell from the Mitocheck dataset [54] are shown in Figure 10. Due to space limitations, multiple single-cell tracking results are combined to be shown in Figure 10. It is obviously seen from Figure 10 that the low-quality (low-contrast) images, illumination variations, and large intensity variations challenge the cell tracking methods. Due to the powerful representation learned from multilayer CDBNs with local tied weights to reduce the model complexity under the scarcity of training samples, our method can still provide promising single-cell tracking results.

## 6. Conclusion

In this paper, we have proposed a robust single-cell/object tracking method via learning and transferring CDBN features. The proposed CDBNTracker does not rely on engineered features and automatically learns the most discriminative features in a data-driven way. A simple yet effective method has been used to transfer the generic and midlevel features learned from CDBNs to the single-cell/object tracking task. The drifting problem is alleviated by exploiting ground-truth, long-term, and short-term positive samples. Extensive experiments on the Mitocheck cell dataset and CVPR2013 tracking benchmark have validated the robustness and effectiveness of the proposed CDBNTracker.

## Figures and Tables

**Table 1:** A representative success score (AUC) of SRE for different subsets divided based on main variation of the target object. Only the top 5 trackers are displayed for clarity.

Image attributes	Ranking				
	The first	The second	The third	The fourth	The fifth
Fast motion (17)	CDBN-10-2 (0.472)	Struck (0.451)	TLD (0.385)	CXT (0.348)	OAB (0.322)
Background clutter (21)	CDBN-10-2 (0.414)	ASLA (0.410)	Struck (0.408)	SCM (0.387)	VTD (0.377)
Motion blur (12)	CDBN-10-2 (0.530)	Struck (0.452)	TLD (0.392)	CXT (0.354)	DFT (0.325)
Deformation (19)	CDBN-10-2 (0.451)	Struck (0.398)	ASLA (0.386)	DFT (0.364)	CPF (0.362)
Illumination variation (25)	CDBN-10-2 (0.440)	ASLA (0.405)	Struck (0.396)	SCM (0.389)	VTS (0.378)
In-plane rotation (31)	CDBN-10-2 (0.422)	CXT (0.410)	Struck (0.410)	ASLA (0.405)	SCM (0.399)
Low resolution (4)	CDBN-10-2 (0.387)	Struck (0.360)	MTT (0.326)	OAB (0.311)	TLD (0.305)
Occlusion (29)	CDBN-10-2 (0.441)	Struck (0.405)	SCM (0.398)	TLD (0.384)	LSK (0.384)
Out-of-plane rotation (39)	CDBN-10-2 (0.427)	Struck (0.409)	ASLA (0.404)	SCM (0.396)	VTD (0.392)
Out of view (6)	CDBN-10-2 (0.457)	Struck (0.421)	LOT (0.411)	TLD (0.407)	CPF (0.394)
Scale variation (28)	CDBN-10-2 (0.441)	ASLA (0.440)	SCM (0.438)	Struck (0.395)	TLD (0.384)
